# Classification of the European Union member states according to the relative level of sustainable development

**DOI:** 10.1007/s11135-015-0278-x

**Published:** 2015-10-23

**Authors:** Bluszcz Anna

**Affiliations:** 1 Faculty of Mining and Geology, Silesian University of Technology, Akademicka 2a, 44-100 Gliwice ul, Poland; 2 ul. Pawła 17/5, 41-708 Ruda Śląska, Poland

**Keywords:** Sustainable development, Taxonomy, Synthetic index

## Abstract

Nowadays methods of measurement and assessment of the level of sustained development at the international, national and regional level are a current research problem, which requires multi-dimensional analysis. The relative assessment of the sustainability level of the European Union member states and the comparative analysis of the position of Poland relative to other countries was the aim of the conducted studies in the article. EU member states were treated as objects in the multi-dimensional space. Dimensions of space were specified by ten diagnostic variables describing the sustainability level of UE countries in three dimensions, i.e., social, economic and environmental. Because the compiled statistical data were expressed in different units of measure, taxonomic methods were used for building an aggregated measure to assess the level of sustainable development of EU member states, which through normalisation of variables enabled the comparative analysis between countries. Methodology of studies consisted of eight stages, which included, among others: defining data matrices, calculating the variability coefficient for all variables, which variability coefficient was under 10 %, division of variables into stimulants and destimulants, selection of the method of variable normalisation, developing matrices of normalised data, selection of the formula and calculating the aggregated indicator of the relative level of sustainable development of the EU countries, calculating partial development indicators for three studies dimensions: social, economic and environmental and the classification of the EU countries according to the relative level of sustainable development. Statistical date were collected based on the Polish Central Statistical Office publication.

## Introduction

Today there are many definitions of sustainable development in literature, because as a principle it is a multi-dimensional problem, hence the differences in stressing the most important issues.

Generally, sustainable development concerns achieving balance in three main dimensions at once, i.e., in the economic dimension signifying the pursuit of sustainable economic development; in the social dimension signifying the protection of public health and social integration; and in the environmental dimension placing a significant emphasis on environmental protection and natural resources in a way as not to endanger the capabilities to meet the needs of future generations (Bluszcz and Kijewska 2015; Kijewska 2016; Fleurbaey 2015; Kates et al. 2005; WCED 1987, Strange and Bayley 2008).

Environmental sustainability could be defined more precisely as a condition of balance, resilience and interconnectedness that allows human society to satisfy its needs while neither exceeding the capacity of its supporting ecosystems to continue to regenerate the services necessary to meet those needs nor by our actions diminishing biological diversity (Morelli 2011).

The environmental dimension relates primarily to the protection of resources (Dubiński and Turek 2014; Dubinski 2013; Wrana et al. 2014) and minimising the negative impacts of industrial activity of the human (Gajdzik 2009, 2012a, b; Gajdzik and Wycislik 2012; Klosok-Bazan et al. 2014; Rogulski and Badyda 2015; Krzemien et al. 2013).

The ecological sustainability term is popularised in literature which connects human needs without compromising the health of ecosystems (Callicott and Mumford 1997).

Currently the economic dimension of sustainable development is a major problem of the economy and is an important trend of economic research (Dunford and Smith 2000; Domanski 2005, 2010; Ziemianczyk 2010, Schultmann et al. 2001; Barro and Sala-i-Martin 2004; Hąbek and Wolniak 2015, Habek 2012, 2014; Epstein 2008; Spence 2009).

Economic growth is one of the most important policy goals across the world, commonly accepted by the society (Moldan et al. 2012).

The social dimension relates primarily to the standard of living of the population, demographic changes and issues of health protection (Stec et al. 2014).

Social Sustainability according to McKenzie is defined as: a positive condition within communities, and a process within communities that can achieve that condition. This definition is supplemented with a list of corresponding principles, including for example: equity of access to key services, equity between generations, a system of relations valuing disparate cultures, political participation of citizens, particularly at a local level, a sense of community ownership (McKenzie 2004).

The member countries in the European Union are characterised by a significant variation in terms of the sustainable development level. Disparities in this area can adversely affect the functioning of the economic system. It can be observed, in recent years the division in regions characterised by the dynamic development and hence regions strongly differentiating from this level has occurred (Wójcik 2008).

The main challenges for the strategy of the European Union are the existing differences in the development level in regions of Europe, including in particular the newly accepted countries (Fourth Report, 2007; Territory matter 2006).

In this situation it becomes very important to continue to monitor the changes in the level of development of countries and hence results the significant number of publications concerning the methodological and empirical studies in this area (Rodriguez-Lopez et al. 2009; Henley 2005; Kosfeld et al. 2006; Petrakos 2001; Royuela and Artis 2006; Burchart-Korol et al. 2014).

The European Union has developed methods of continuous monitoring of progress towards achieving the sustainable development of the member states based on the specified main indicators concerning the following issues: socio-economic development, sustainable consumption and production, social inclusion, demographic changes, public health, climate change and energy, sustainable transport, natural resources, global partnership, good governance (Sustainable development report 2014, Analysis of Innovation report 2011).

These methods are based on the analysis and assessment of diversified partial indicators.

The article presented a different approach to the topic of assessing the level of sustainable development, involving its comprehensive assessment.

The aim of the studies conducted in the article was the relative assessment of the sustainable development level of the European Union member countries and the comparative analysis of the Poland position relative to other EU member states. For the realisation of thus defined aim the quantitative methods were applied, which allow the objectification of the conducted comparative analyses and research, based on figures characterising the situation of the studied countries in areas concerning three dimensions of sustainable economic, environmental and social development. Statistical data were collected from ten diagnostic variables such as: population density persons per km^2^; deaths per 1000 population; infant deaths per 1000 live births; natural increase per 1000 population; employment rate of persons aged 15–64 in %; labour productivity; debt of the general government sector in % GDP; GDP per capita at purchasing power parity; greenhouse gas emissions; total production of primary energy per capita in TOE.

The level of sustainable development was defined based on the aggregation of the listed diagnostic variables into one synthetic indicator, which enabled the normalised measurement of development of the studied EU countries. Another important aspect of studies was to determine the degree of diversity of the levels of sustainable development between member countries, which was assessed using the variability indicator of the aggregated development measure. Also the comparative analyses were presented in partial dimensions independently economic, social and environmental.

## Literature review

The Sustainable Development Indicators (SDIs) are used to monitor the EU Sustainable Development Strategy (EU SDS) in a report published by Eurostat every two years. Of more than 100 indicators, eleven have been identified as headline indicators. They are intended to give an overall picture of whether the European Union has achieved progress towards sustainable development in terms of the objectives and targets defined in the strategy (EUROSTAT 2015).

In addition to partial indicators many other synthetic indicators have been developed, which had a more general character. Synthetic indicators describe and measured the whole processes constituting the quality of life of the population and the total effect of mutual impact of the economic sphere and the environment. These synthetic indicators can include, among others: The Sustainable Society Index (SSI), The Environmental Performance Index (EPI), Index of Economic Well-being, The Environmental Sustainability Index (ESI).

The SSI is a synthetic measure developed by the Sustainable Society Foundation. It expresses the level of balance between the examined countries as well as the distance separating them from the pre-defined desired state in accordance with a group of analysed indices. The SSI was first published in 2006 and its magnitude fits into the normalized scope of [0;10]. The SSI covers 151 countries, comprising no less than 99 % of the world population. It is built up by 21 indicators, clustered in 7 categories such as: basic needs, health, personal and social development, natural resources, climate and energy, transition and economy and finally connected into 3 main dimensions: Human Well-being; Environmental Well-being and Economic Well-being (Van Kerk and Manuel 2014; SSI Rankings 2014).

The EPI measuring the ecological level of countries was devised at the American Universities of Columbia and Yale. The synthetic index was normalized within the scale of [0;100]. The first analysis took place in 2006 and comprised 146 countries. The EPI is constructed through the calculation and aggregation of 20 indicators reflecting national-level environmental data. These indicators are combined into nine issue categories such as health impacts, air quality, water and sanitation, water resources, agriculture, forests, fisheries, biodiversity and habitat, climate and energy, each of which fit under one of two overarching objectives Environmental Health and Ecosystem Vitality (EPI 2013).

In 1998 the Centre for the Study of Living Standards developed the Index of Economic Well-being, based on a paper written by Lars Osberg for the MacDonald Commission entitled The Measurement of Economic Welfare. It comprises the following four domains of economic well-being:

1. Effective per capita consumption flows, including consumption of marketed goods and services; government services; effective per-capita flows of household production; leisure; and changes in life span.
2. Net societal accumulation of stocks of productive resources, including net accumulation of tangible capital; housing stocks; net changes in the value of natural resources stocks; environmental costs; net changes in the level of foreign indebtedness; accumulation of human capital; and the stock of R&D investment.
3. Income distribution, including the intensity of poverty (incidence and depth) and the inequality of income.
4. Economic security from job loss and unemployment, illness, family breakup and poverty in old age (Osberg and Sharpe 2001; IEWB 2015).

The ESI is a measure of overall progress towards environmental sustainability. As a composite index it tracks a set of environmental, socioeconomic, and institutional indicators that characterize and influence environmental sustainability at a national level. The ESI is part of a large project called The Environmental Performance Measurement (EPM) project, an initiative of the Yale Center for Environmental Law and Policy (YCELP) and the Center for International Earth Science Information Network (CIESIN) of Columbia University, in collaboration with the World Economic Forum and the Joint Research Centre (JRC) of the European Commission (ESI 2015).

Comparative analyses in the area of the sustainability level of EU member states were conducted by, among others: Venkatesh using Total Sustainability Index (TSI) to compare development of twelve Asian countries (Venkatesh 2015); Bujnowicz-Haraś et al. performed the assessment of the level of sustainable development of countries of the European Union using taxonomic methods in the form of the Hellwing development model built based on 23 variables grouped into 6 thematic groups such as: socioeconomic development, sustainable consumption and production, social inclusion, demographic changes, public health, climate change and energy, natural resources, sustainable transport (Bujnowicz-Haras et al. 2015); Żelazna and Gołębiowska conducted studies of monitoring indicators related to energy in the member countries in particular within the scope of: greenhouse gas emissions, share of renewable energy in gross final energy consumption and primary energy consumption (Zelazna and Golebiowska 2015); Stec et al. performed the assessment of socio-economic aspects of sustainable development in the EU countries using 27 variables in four groups: demographic potential and labour market, economic potential, level of development of social infrastructure and level of development of technical infrastructures (Stec et al. 2014); Bringezy conducted studies about the use of materials in the industry of the EU member states (Bringezu 2002).

The presented methodologies of sustainable measurement do not exhaust the multi-dimensional subject, and only confirm the fact that there is no single, universal measurement tool comprising the many aspects of sustainable development. Hence results the continuous topicality of the research issue undertaken in the article.

## Research design

Implementation of the research goal assumed in the paper consisting of the relative assessment of the sustainability level of EU member states was conducted using the aggregated indicator of the relative sustainability level covering ten diagnostic variables characterising sustainable development of the EU member countries. The comparative analysis was conducted based on the linear ordering of countries according to the aggregated indicator and based on the volatility index.

The research methodology algorithm included 8 stages:

1. Defining data matrices,
2. Calculating the variation index for all variables and elimination of variables, which coefficient is less than 10 %,
3. Division of variables into stimulants and de-stimulants,
4. Selection of the method of zero unitarisation for transforming variables,
5. Developing normalised data matrices,
6. Calculating the aggregated indicator of the relative level of sustainable development of the EU countries,
7. Calculating indicators of development for three studied dimensions: social, economic and environmental,
8. Classification of the EU countries according to the relative level of sustainable development.

## Sustainability level of European Union member states

The relative level of sustainable development of EU member states was achieved according to eight stages in this research.

In the first research stage the process of defining the scope of entry data was necessary to assess the relative level of sustainable development of the European Union countries. Based on statistical data of Eurostat ten variables were used, which characterised the socio-demographic, economic and environmental situation of the studied member countries. Statistical data cover the 2012 year and were presented in Table 1.

**Table 1:** Diagnostic variables in three dimensions: social, economic and environmental

Diagnostic variable		
Diagnostic variable in human dimension		
X1	Destimulant	Population density persons/km^2^
X2	Destimulant	Deaths per 1000 population
X3	Destimulant	Infant deaths per 1000 live births
X4	Stimulant	Natural increase per 1000 population
X5	Stimulant	Employment rate of persons aged 15–64 (in %)
Diagnostic variable in economic dimension		
X6	Stimulant	Labour productivity (EU-27 = 100)
X7	Destimulant	Debt of the general government sector in % GDP
X8	Stimulant	GDP per capita at purchasing power parity (current prices) EU-28 = 100
Diagnostic variable in environmental dimension		
X9	Destimulant	Greenhouse gas emissions (1990 = 100)
X10	Destimulant	Total production of primary energy per capita in TOE

Data covering three dimensions of sustainable development, i.e., the social, economic and environmental areas were collected for analysis. The first group included the potential diagnostic variables describing the socio-demographic situation of the studied member countries, which concern the most important thematic areas, such as living conditions of the population (population density persons/km^2^ and deaths per 1000 population), demographic changes characterising the percentage of people in the working age (employment rate of persons aged 15–64 in %) and public health described using the following criteria (Infant deaths per 1000 live births and deaths per 1000 population).

The economic dimension was characterised based on three potential diagnostic variables, i.e.: debt of the general government sector in % GDP, labour productivity (EU-27 = 100) and GDP per capita at purchasing power parity (current prices) EU-28 = 100.

While the environmental dimension was described by two potential diagnostic variables such as: Greenhouse gas emissions and total production of primary energy per capita in TOE.

Potential diagnostic variables adopted for analysis should be characterised by the significant variability, interpreted as the ability to diversify the studied countries among them, hence results the second stage of studies.

In the second stage of research the variability indicator *V*_*j*_ was calculated as the ratio of standard deviation and the arithmetic mean for each potential diagnostic variable. Then the elimination should take place from the further analysis of these variables, which variability coefficient is less than 10 %. Calculations were performed based on formulas 1, 2, 3 while results of calculations were presented in Table 2.

1 $$V_{j} = \frac{{S_{j}^{x} }}{{\overline{x} }} \cdot 100$$

where:

2 $$S_{j}^{x} = \sqrt {\frac{1}{n}\sum\limits_{i = 1}^{n} {(x_{j} - \bar{x}_{j} } } )^{2}$$

3 $$\overline{{x_{j} }} = \frac{1}{n}\sum\limits_{i = 1}^{n} {x_{j} }$$

**Table 2:** Coefficients of variation for diagnostic variables

	X1	X2	X3	X4	X5	X6	X7	X8	X9	X10
$S_{j}^{x}$	262.90	3.79	1.95	4.27	20.57	37.86	40.40	49.79	37.70	1.09
$\bar{x}$	173.79	9.22	3.76	1.04	55.73	82.49	61.85	86.23	78.24	1.34
*V*_*j*_ (%)	151.4	21.3	41.1	1034.6	10.4	28.1	52.0	42.3	31.3	76.5

All potential diagnostic variables were characterised by a significant indicator of variability. The lowest level of volatility (10.4 %) was observed for feature 5, which defines the percentage of people employed in the age from 15 to 64, what means the lowest diversity of member countries in this regard. The highest level of variability was achieved by feature 5 defining the level of natural growth (1034 %). This means the greatest diversity of member countries in this regard. It should be noted that all studied features have a significant indicator of volatility over 10 % and were included in the further part of the analysis.

The third stage of the analysis involved the division of variables into stimulants and de-stimulants. Stimulants are variables—features, which growth of values indicates the desired development of the studied phenomenon. Destimulants are variables, which decrease of values means the desired development of the studied phenomenon (Strahl 1984).

The fourth stage of the analysis involved the normalisation of data by using the method of zero unitarisation, which was conducted based on formulas 4 and 5.

Transformation formula for stimulants (Wiszniewska 2008):

4 $$_{{z_{ij} }} = \frac{{x_{ij} - \mathop {\hbox{min} }\limits_{i} \left\{ {x_{ij} } \right\}}}{{\mathop {\hbox{max} }\limits_{i} \left\{ {x_{ij} } \right\} - \mathop {\hbox{min} }\limits_{i} \left\{ {x_{ij} } \right\}}}$$

Transformation formula for destimulants (Wiszniewska 2008):

5 $$_{{z_{ij} }} = \frac{{\mathop {\hbox{max} }\limits_{i} \left\{ {x_{ij} } \right\} - x_{ij} }}{{\mathop {\hbox{max} }\limits_{i} \left\{ {x_{ij} } \right\} - \mathop {\hbox{min} }\limits_{i} \left\{ {x_{ij} } \right\}}}$$

where x_*ij*_ is the value of the diagnostic variable and z_*ij*_ is the normalized value of *x*_*ij*_.

The fifth stage of the analysis included the normalisation of data, which aims to leading variables with different measures to mutual comparability (additivity) and unification of the feature nature, that is replacing diverse scopes of variability of features with a constant. The main aim of standardisation is the elimination of the impact of measure units by introducing additivity in feature sets with different embodiments, that is converting the absolute values into relative values. The developed normalised data matrix based on formulas 4 and 5 was presented in Table 3.

**Table 3:** Matrix of normalised data

Country	X1	X2	X3	X4	X5	X6	X7	X8	X9	X10
France	0.937	0.724	0.743	0.620	0.541	0.607	0.453	0.282	0.583	0.487
Spain	0.944	0.736	0.797	0.440	0.193	0.547	0.482	0.222	0.233	0.821
Sweden	0.996	0.609	0.865	0.513	0.947	0.591	0.807	0.366	0.610	0.026
Germany	0.842	0.483	0.770	0.207	0.906	0.529	0.516	0.352	0.718	0.615
Finland	1.000	0.621	0.892	0.460	0.766	0.546	0.702	0.315	0.511	0.179
Poland	0.922	0.575	0.595	0.367	0.369	0.246	0.689	0.093	0.597	0.538
Italy	0.864	0.540	0.824	0.280	0.250	0.549	0.203	0.245	0.523	0.872
United Kingdom	0.827	0.701	0.662	0.620	0.795	0.468	0.464	0.269	0.715	0.538
Romania	0.948	0.264	0.000	0.187	0.361	0.055	0.809	0.014	0.942	0.641
Greece	0.949	0.517	0.824	0.267	0.025	0.401	0.000	0.130	0.390	0.769
Bulgaria	0.962	0.000	0.162	0.000	0.332	0.000	0.941	0.000	0.849	0.590
Hungary	0.932	0.230	0.554	0.107	0.266	0.226	0.524	0.093	0.786	0.718
Portugal	0.926	0.552	0.757	0.253	0.455	0.266	0.223	0.134	0.325	0.897
Croatia	0.975	0.333	0.730	0.207	0.000	0.306	0.689	0.065	0.581	0.795
Austria	0.938	0.644	0.784	0.360	0.893	0.596	0.564	0.384	0.408	0.615
Czech Republic	0.914	0.540	0.865	0.367	0.648	0.249	0.753	0.157	0.775	0.231
Ireland	0.965	1.000	0.743	1.000	0.332	0.824	0.269	0.380	0.425	0.923
Lithuania	0.976	0.149	0.689	0.133	0.463	0.249	0.791	0.116	1.000	0.897
Latvia	0.987	0.080	0.365	0.067	0.504	0.183	0.791	0.079	0.996	0.718
Slovakia	0.931	0.609	0.432	0.407	0.369	0.317	0.710	0.134	0.823	0.692
Estonia	0.990	0.379	0.730	0.293	0.672	0.215	1.000	0.111	0.930	0.026
Denmark	0.917	0.644	0.757	0.433	0.898	0.572	0.758	0.366	0.635	0.128
Netherlands	0.718	0.759	0.716	0.507	1.000	0.541	0.582	0.370	0.529	0.000
Belgium	0.756	0.598	0.703	0.480	0.455	0.711	0.388	0.338	0.619	0.641
Slovenia	0.938	0.644	1.000	0.453	0.549	0.308	0.807	0.171	0.424	0.564
Cyprus	0.948	0.966	0.743	0.713	0.570	0.414	0.478	0.208	0.036	0.974
Luxembourg	0.871	0.885	0.878	0.633	0.619	1.000	0.919	1.000	0.478	0.949
Malta	0.000	0.793	0.500	0.480	0.340	0.403	0.582	0.181	0.000	1.000

The sixth step included calculating the aggregated indicator relative to the level of sustainable development of the EU countries. The aggregate indicator was calculated as the non-weighed arithmetic mean of normalised values according to formula 6.

6 $$Wsd_{i} = \frac{1}{m}\sum\limits_{j = 1}^{m} {z_{ij} } \quad i = 1,2,\ldots,n$$

Value of aggregate indicators *Wsd*_*i*_ will create a vector

7 $$Wsd_{{_{i} }} = \begin{array}{*{20}c} {Wsd_{1} } \\ {Wsd_{2} } \\ \vdots \\ {Wsd_{n} } \\ \end{array}$$

The higher the value of the aggregate synthetic indicator, the higher the level of sustainable development of the studied member countries from the point of view of diagnostic variables included in the analysis. Results of calculations were presented in Table 4.

**Table 4:** Position of the EU member in the ranking according the synthetic indicator

Position of the EU member		*Wsd*_*i*_	Position of the EU member		*Wsd*_*i*_
1	Luxembourg	0.8232	15	Lithuania	0.5465
2	Ireland	0.6860	16	Slovakia	0.5425
3	Sweden	0.6330	17	Spain	0.5414
4	Austria	0.6187	18	Estonia	0.5346
5	Denmark	0.6106	19	Italy	0.5152
6	United Kingdom	0.6059	20	Poland	0.4989
7	Cyprus	0.6050	21	Portugal	0.4788
8	Finland	0.5993	22	Latvia	0.4770
9	France	0.5978	23	Croatia	0.4682
10	Germany	0.5938	24	Hungary	0.4435
11	Slovenia	0.5858	25	Malta	0.4279
12	Netherlands	0.5722	26	Greece	0.4272
13	Belgium	0.5688	27	Romania	0.4221
14	Czech Republic	0.5497	28	Bulgaria	0.3835

Poland was classified on the twentieth location in the ranking according to the level of sustainable development of member countries. The level of the indicator for Poland was 0.4989, because the arithmetic mean of the aggregated indicator of sustainable development was 0.5485, this means that countries up to the 14 position in the ranking, including, (that is from Luxembourg to the Czech Republic) are characterised by the high level of sustainable development, while countries from the 15 position in the ranking are characterised by the sustainable development under the mean level achieved by the EU member countries. The leader in the ranking is Luxembourg, which reached the highest level of the aggregated indicator (0.8232), while the country with the lowest level of the sustainable indicator of development is Bulgaria (0.3835). The variability index (formula 1) of the aggregated indicators of sustainable development was 16.8 % what means a considerable diversification in terms of ten studied diagnostic variables.

**Fig. 1:**
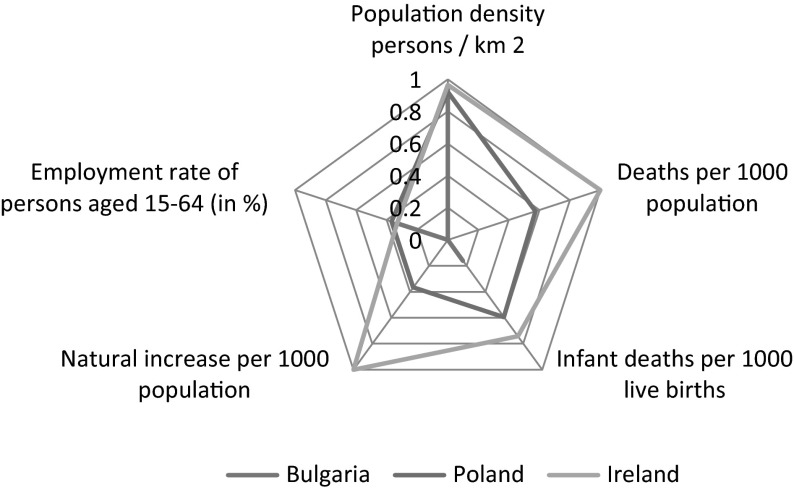
Human dimension

**Fig. 2:**
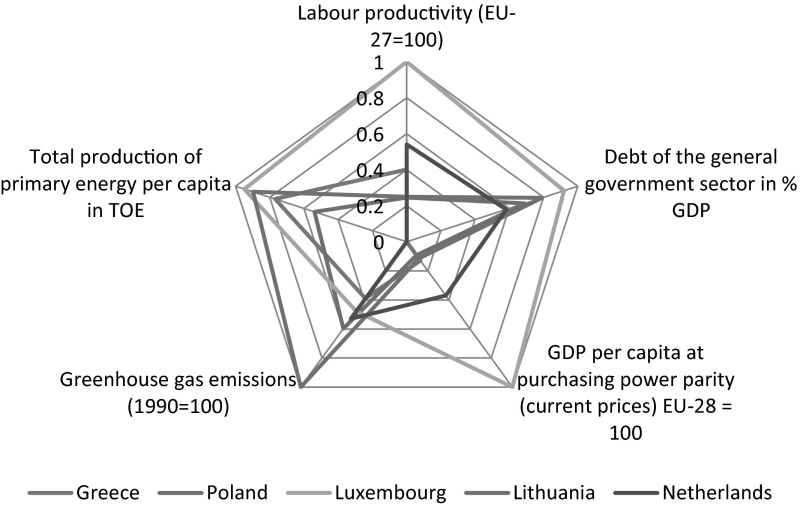
Economic and environmental dimensions

The seventh stage concerned calculation of development indicators for three studied dimensions: social, economic and environmental separately. Appropriate calculations according formula 6 were presented in Table 5 and in Figs. 1 and 2.

**Table 5:** Position of the European Union member states in the three dimensions: social, economic and environmental separately

Human dimension			Economic dimension			Environmental dimension		
1	Ireland	0.8080	1	Luxembourg	0.9730	1	Lithuania	0.9487
2	Cyprus	0.7880	2	Sweden	0.5880	2	Latvia	0.8571
3	Sweden	0.7860	3	Denmark	0.5651	3	Romania	0.7915
4	Luxembourg	0.7774	4	Finland	0.5211	4	Slovakia	0.7578
5	Finland	0.7478	5	Austria	0.5147	5	Hungary	0.7520
6	Netherlands	0.7400	6	Netherlands	0.4978	6	Bulgaria	0.7192
7	Denmark	0.7296	7	Ireland	0.4906	7	Luxembourg	0.7131
8	Austria	0.7238	8	Belgium	0.4790	8	Italy	0.6976
9	United Kingdom	0.7211	9	Germany	0.4657	9	Croatia	0.6881
10	Slovenia	0.7168	10	France	0.4477	10	Ireland	0.6742
11	France	0.7130	11	Estonia	0.4422	11	Germany	0.6666
12	Czech Republic	0.6667	12	Slovenia	0.4287	12	Belgium	0.6299
13	Germany	0.6415	13	Spain	0.4171	13	United Kingdom	0.6267
14	Spain	0.6219	14	United Kingdom	0.4001	14	Portugal	0.6113
15	Estonia	0.6129	15	Malta	0.3885	15	Greece	0.5795
16	Belgium	0.5982	16	Slovakia	0.3873	16	Poland	0.5678
17	Portugal	0.5884	17	Czech Republic	0.3862	17	France	0.5352
18	Poland	0.5653	18	Lithuania	0.3855	18	Spain	0.5266
19	Italy	0.5518	19	Cyprus	0.3667	19	Austria	0.5119
20	Slovakia	0.5495	20	Croatia	0.3534	20	Cyprus	0.5049
21	Greece	0.5164	21	Latvia	0.3508	21	Czech Republic	0.5028
22	Lithuania	0.4821	22	Poland	0.3424	22	Malta	0.5000
23	Croatia	0.4490	23	Italy	0.3325	23	Slovenia	0.4942
24	Malta	0.4227	24	Bulgaria	0.3136	24	Estonia	0.4778
25	Hungary	0.4178	25	Romania	0.2927	25	Denmark	0.3814
26	Latvia	0.4006	26	Hungary	0.2808	26	Finland	0.3454
27	Romania	0.3520	27	Portugal	0.2079	27	Sweden	0.3180
28	Bulgaria	0.2912	28	Greece	0.1769	28	Netherlands	0.2645

Due to five diagnostic variables characterising the social dimension of sustainable development, i.e.: population density persons/km^2^; deaths per 1000 population; employment rate of persons aged 15–64 in %; infant deaths per 1000 live births and deaths per 1000 population Poland reached the eighteenth location in the ranking of the EU countries with the aggregated index at the level of 0.5653. The arithmetic mean of the level of indicators of social dimensions of the EU countries was 0.6064, what means that 15 countries were classified at the level over the mean and that 13 countries reached the level of social development under the European mean and this group, unfortunately, included Poland. The highest level of the aggregated indicator in this dimension was obtained by Ireland (0.8080), and the lowest level was achieved by Bulgaria (0.2912).

The economic dimension was characterised based on three potential diagnostic variables, i.e.: debt of the general government sector in % GDP, labour productivity (EU-27 = 100) and GDP per capita at purchasing power parity (current prices) EU-28 = 100.

In the assessment of the economic dimension Poland took the twenty-seventh location achieving the level of the aggregated economic measure of 0.3424. The mean level of the economic measure of the EU countries was 0.4213, what means that the level of the economic development of Poland is under the mean level of the EU countries. The best economic situation is shown by Luxembourg (0.9730), and the worst one by Greece (0.1769).

The environmental dimension was described by two potential diagnostic variables, i.e.: Greenhouse gas emissions and total production of primary energy per capita in TOE.

Analysing the environmental aspects Poland was classified on the sixteenth position in the ranking (0.5678). The average level of the environmental measure of the EU countries was 0.5944. The leader in this dimension is Lithuania (0.9487) and the last position is taken by the Netherlands (0.2645).

The data analysis has also shown that the member countries are the most diverse in the economic dimension, as the level of the variability indicator for the aggregated measure was as much as 34.6 %. The variability indicator of the aggregated measure of the social dimension was 24.08 % while of the environmental dimension 27.19 %, what should be interpreted as a significant diversity of countries in these dimensions among themselves. Results of the comparative analysis were also presented in charts 1 and 2.

The Figs. 1 and 2 also present the comparison of analysis results in three dimensions of sustainable development: social, economic and environmental. The charts present the position of Poland in reference to the best and the worst member country according to the level of sustainable development in each of the three dimensions.

**Fig. 3:**
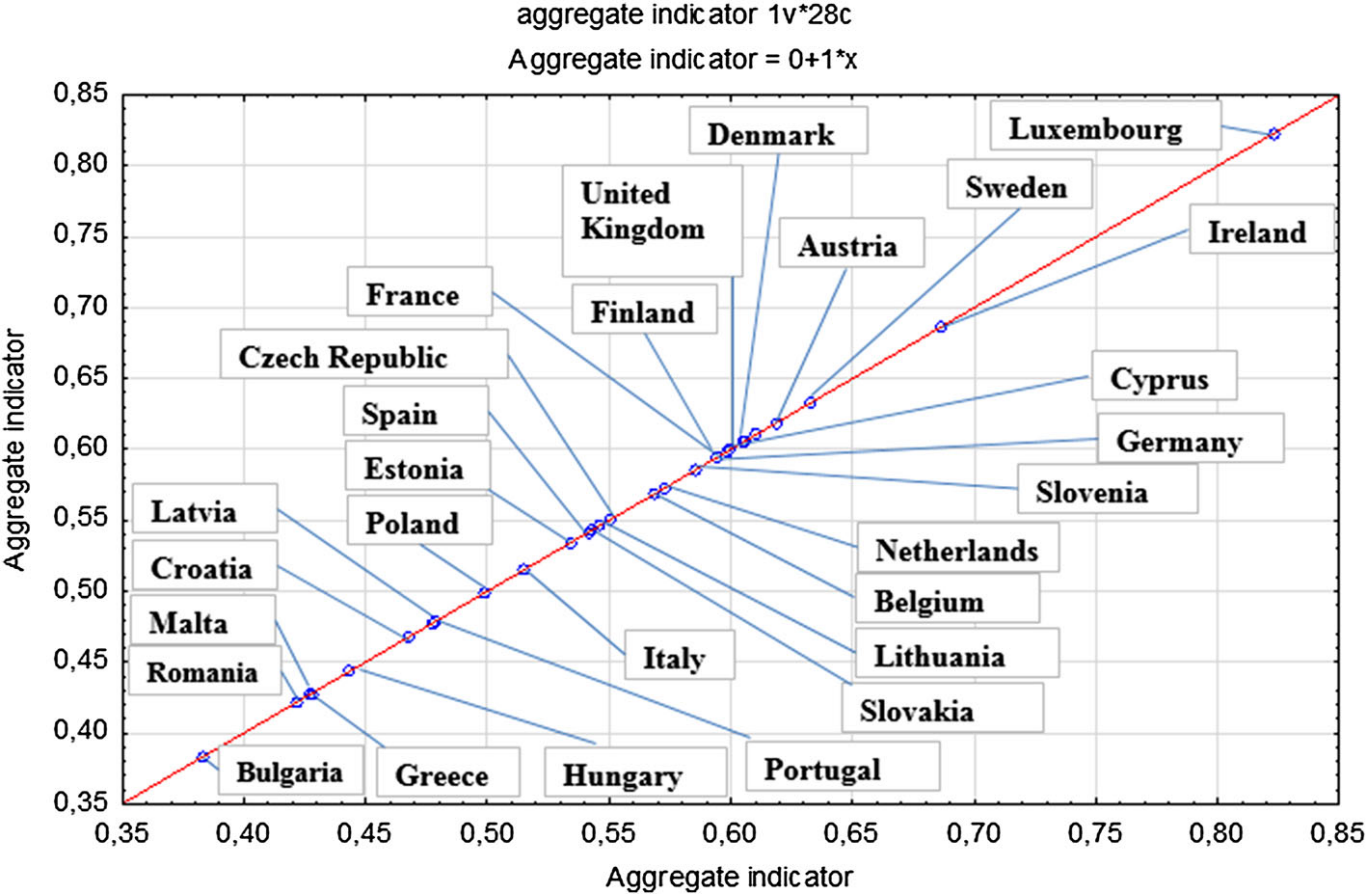
Ranking of the European Union countries according to the level of sustainable development

The last eight stage of the study concerned the classification of the European Union countries according to the level of sustainable development. The linear organisation of the EU countries according to the aggregated indicator was presented in Table 4 and in Fig. 3.

The analysis covered 28 member countries of the European Union in terms of ten diagnostic variables presented in Table 6, which characterise the level of sustainable development of countries in three dimensions: social, economic and environmental. The analysis was conducted based on the statistical data for 2012. Comparing the level of sustainable development took place based on the aggregated indicator of sustainable development. The highest level of development was achieved by Luxembourg (0.8232), which has significantly gone ahead of other countries, such as: Ireland (0.6860), Sweden (0.6330) and Austria (0.6187). Poland took the middle position due to the level of the aggregated indicator (0.4989). The last places in the ranking were taken by Greece (0.4272), Romania (0.4221) and Bulgaria (0.3835).

**Table 6:** Sustainable development indicators

Theme	Headline indicator
Socio-economic development	Growth rate of actual GDP per capita
Sustainable consumption and production	Resource productivity
Social inclusion	Persons at-risk-of-poverty or social exclusion
Demographic changes	Employment rate of older workers
Public health	Healthy life years and life expectancy at birth, by sex
Climate change and energy	Greenhouse gas emissions Share of renewable energy in gross final energy consumption Primary energy consumption
Sustainable transport	Energy consumption of transport relative to GDP
Natural resource	Common bird index
Global partnership	Official development assistance as share of gross national income
Good governance	No headline indicator

*Source* http://ec.europa.eu/eurostat/web/sdi/indicators. Accessed 20 June 2015

## Conclusions

Comparative studies are an important tool in the process of implementing the ideas of sustainable development. Monitoring the spatial diversity of the development of member countries is the source of valuable information for determining strategic actions and determining future trends. Determining the position of the studied countries in terms of each other enables the performance of observations in time and thus the assessment of the occurring changes.

The potential set of indicators describing sustainable development of the studied EU countries included ten diagnostic variables, i.e. population density persons per km^2^; deaths per 1000 population; infant deaths per 1000 live births; natural increase per 1000 population; employment rate of persons aged 15–64 in  %; labour productivity; debt of the general government sector in % GDP; GDP per capita at purchasing power parity; greenhouse gas emissions; total production of primary energy per capita in TOE.

Studies undertaken in the paper within the assessment of the level of sustainable development of member countries do not exhaust the multi-dimensional issues however, they are a crucial addition of the analyses undertaken in the literature. It should be generally stated that Poland is a country approaching the mean level of sustainable development of member countries. Poland took the sixteenth position in the ranking in the environmental dimension. In the social dimension Poland took the eighteenth position, while in the economic dimension Poland was classified on the twenty-second position. According to the aggregated relative measure of sustainable development Poland took the twentieth position.

## References

[CR1] Analysis of innovation drivers and barriers in support of better policies Economic and Market Intelligence on Innovation Integrated Innovation Policy for an Integrated Problem: Addressing Climate Change, Resource Scarcity and Demographic Change to 2030 Final Report. European Commission (2011)

[CR2] Barro R. J. and Sala-i-Martin X.: Economic growth, 2nd edn. The MIT Press Cambridge, London. http://down.cenet.org.cn/upfile/8/200751171644184.pdf (2004). Accessed 20 June 2015

[CR3] Bluszcz A, Kijewska A (2015). Challenges of sustainable development in the mining and metallurgy sector in Poland. Metalurgija.

[CR4] Bringezu S.: Towards Sustainable resource management in the European Union. Wuppertal Paper 121, Wuppertal Institute for Climate, Environment, Energy 121 (2002)

[CR5] Bujnowicz-Haraś B, Janulewicz P, Nowak A, Krukowski A (2015). Evaluation of sustainable development in the member states of the European Union. Probl. Sustain. Dev..

[CR6] Burchart-Korol D, Krawczyk P, Czaplicka-Kolarz K, Turek M, Borkowski W (2014). Development of sustainability assessment method of coal mines. J. Sustain. Min..

[CR7] Callicott JB, Mumford K (1997). Ecological sustainability as an conservation concept. Conserv. Biol..

[CR8] Domański B (2005). The economic performance and standard of living of post-communist European countries since 1989: factors and processes behind. Geogr. Polonica.

[CR9] Domański B (2010). Should we fight local and regional disparities in economic development in Poland?. Studia Regionalia KPZK PAN.

[CR10] Dubiński J (2013). Zrównoważony rozwój górnictwa surowców mineralnych. J. Sustain. Min..

[CR11] Dubiński J, Turek M (2014). Chances and threats of hard coal mining development in Poland—the results of experts research. Arch. Min. Sci..

[CR12] Dunford M, Smith A (2000). Catching up or falling behind? Economic performance and regional trajectories in the “New Europe”. Econ Geogr.

[CR13] Environmental Performance Index (EPI): http://epi.yale.edu/ Sustainable Development in the European Union: 2013 monitoring report of the EU sustainable development strategy. European Commission. Eurostat (2013). http://ec.europa.eu/eurostat/documents/3217494/5760249/KS-02-13-237-EN.PDF Accessed 20 June 2015

[CR14] Environmental Sustainability Index (ESI): http://sedac.ciesin.columbia.edu/data/collection/esi/. Accessed 20 June 2015

[CR15] Epstein MJ (2008). Making Sustainability Work. Best Practices in Managing and Measuring Corporate Social, Environmental, and Economic Impacts.

[CR16] ESPON Territory matters for competitiveness and cohesion. Facets of regional diversity and potentials in Europe. European Spatial Planning Observation Network, Synthesis Report III, results by autumn 2006. Luxembourg http://www.espon.eu/export/sites/default/Documents/Publications/ESPON2006Publications/SynthesisReport3/final-synthesis-reportiii_web.pdf (2006) Accessed 20 June 2015

[CR17] EUROSTAT: Sustainable Development Indicators. http://ec.europa.eu/eurostat/web/sdi/indicators (2015). Accessed 20 June 2015

[CR18] European Commission: Fourth report on economic and social cohesion, growing regions, growing Europe. Luxembourg. http://ec.europa.eu/regional_policy/sources/docoffic/official/reports/cohesion4/pdf/4cr_en (2007). Accessed 20 June 2015

[CR19] Fleurbaey M (2015). On sustainability and social welfare. J. Environ. Econ. Manag..

[CR20] Gajdzik B (2009). Environmental aspects, strategies and waste logistic system based on the example of metallurgical company. Metalurgija.

[CR21] Gajdzik B (2012). Comprehensive classification of environmental aspects in a manufacturing enterprise. Metalurgija.

[CR22] Gajdzik B (2012). The ecological value of metallurgical enterprise after privatization and restructuring. Metalurgija.

[CR23] Gajdzik B, Wyciślik A (2012). Assessment of environmental aspects in a metallurgical enterprise. Metalurgija.

[CR24] Hąbek P (2012). Sustainability report. Disclosure the impact of business on society and the environment. Sci. J. Maritime Univ. Szczecin.

[CR25] Hąbek P (2014). Evaluation of sustainability reporting practices in Poland. Springer Quality&Quantity.

[CR26] Hąbek P., Wolniak R.: Assessing the quality of corporate social responsibility reports; the case of reporting practices in selected European Union member states. Springer Quality&Quantity 1–22 (2015) 10.1007/s11135-014-0155-z PMC4705117 26792950

[CR27] Henley A (2005). On regional growth convergence in Great Britain. Reg. Stud..

[CR28] IEWB-Index of Economic Weel -Being http://www.csls.ca/iwb.asp Accessed 20 June 2015

[CR29] Kates RW, Parris TM, Leiserowitz AA (2005). What is sustainable development? Goals, indicators, values, and practice. Environ. Sci. Policy Sustain. Dev..

[CR30] Kijewska A (2016). Conditions for sustainable growth (SGR) for companies from metallurgy and mining sector in Polands. Metalurgija.

[CR31] Kłosok-Bazan I, Gajdzik B, Machnik-Słomka J, Ocieczek W (2014). Environmental aspects of innovation and new technology implementation in metallurgy industry. Metalurgija.

[CR32] Kosfeld R, Eckey HF, Dreger C (2006). Regional productivity and income convergence in Unified Germany 1992–2000. Reg. Stud..

[CR33] Krzemień A, Więckol-Ryk A, Duda A, Koteras A (2013). Risk assessment of a post—combustion and amine—based CO_2_ capture ready process. J. Sustain. Min..

[CR34] McKenzie S., Social Sustainability: Towards some definitions. Hawke Research Institute Working Paper Series 27 (2004)

[CR35] Moldan B, Januskova S, Hak T (2012). How to understand and measure environmental sustainability: indicators and targets. Ecol. Ind..

[CR36] Morelli J (2011). Environmental sustainability: a definition for environmental professionals. J. Environ. Sustain..

[CR37] Olsberg, L., Sharpe, A.: The index of economic well-being: an overview. http://www.csls.ca/iwb/iwb2002-p.pdf (2001). Accessed 20 June 2015

[CR38] Petrakos G (2001). Patterns of regional inequality in transition economics. Eur. Plan. Stud..

[CR39] Rodriguez-Lopez J, Martinez-Lopez D, Romero-Avila D (2009). Persistence of inequalities across the Spanish regions. Pap. Region. Sci..

[CR40] Rogulski M, Badyda A (2015). Analysis of data on emissions on example of opolskie voivodship within context of fees for use of the environment. Pol. J. Environ. Stud..

[CR41] Royuela V, Artis M (2006). Convergence analysis in terms of quality of life in the urban system of Barcelona Province, 1991–2000. Reg. Stud..

[CR42] Schultmann F, Rainer J, Rent O (2001). A methodlogical approach for the economic assessment of best available techniques. Int. J. Life Cycle Assess..

[CR43] Spence C (2009). Social and environmental reporting and the corporate ego. Bus. Strategy Environ..

[CR44] Stec M, Filip P, Grzebyk M, Pierscieniak A (2014). Socio-economic development in the EU member states—concept and classification. Eng. Econ..

[CR49] Strahl D (1984). Metody ekonometryczne w programowaniu rozwoju przemysłu.

[CR45] Strange, T., Bayley, A.: Sustainable development. Linking economy, society, environment. OECD http://www.oecd.org/insights/sustainabledevelopmentlinkingeconomysocietyenvironment.htm (2008). Accessed 20 June 2015

[CR46] Sustainable Society Index (SSI)—Rankings (2014) from http://www.ssfindex.com/results-2014/ranking-all-countries/ Accessed 20 June 2015

[CR47] Van de Kerk G., Manuel, A.: Sustainable Society Index 2014. Sustainable Society Foundation, the Hague, the Netherlands from http://www.ssfindex.com/ssi2014/wp-content/uploads/pdf/SSI2014.pdf. Accessed 20 June 2015

[CR50] Venkatesh G (2015). Sustainable Development as a single measure: case study of some developing Asian Countries. Problems of Sustainable Development..

[CR48] Wiszniewska E.: Taksonomiczna analiza poziomu zrównoważonego rozwoju województw w Polsce. w: Taksonomia 15. Klasyfikacja i analiza danych—teoria i zastosowania. (ed.) Jajuga K., Walesiak M. Wydawnictwo Naukowe Uniwersytetu Ekonomicznego we Wrocławiu. Wrocław 372-373 (2008)

[CR51] Wójcik P (2008). Dywergencja czy konwergencja: Dynamika rozwoju polskich regionów (Divergence or convergence: Development dynamics of the Polish regions). Studia Regionalne i Lokalne.

[CR52] Word Commission on Environment and Development (WCED) (1987). Our Common Future.

[CR53] Wrana K, Trząski L, Głogowska M, Lebek M, Chmielewski W, Szendera W (2014). Model of local sustainable development in the areas of co-occurrence NATURA 2000 sites and Non Energy Mining Industry (NEEI). J. Sustain. Min..

[CR54] Żelazna A, Gołębiowska J (2015). The measures of sustainable development—a study based on the European Monitoring of energy—related indicators. Probl. Sustain. Dev..

[CR55] Ziemiańczyk U (2010). An assessment of socio-economic development of rural and urban-rural communities in the Malopolska province. Infrastruct. Ecol Rural Areas.

